# Associations of ultra-processed food consumption, circulating protein biomarkers, and risk of cardiovascular disease

**DOI:** 10.1186/s12916-023-03111-2

**Published:** 2023-11-03

**Authors:** Huiping Li, Yaogang Wang, Emily Sonestedt, Yan Borné

**Affiliations:** 1 School of Medicine, Northwest University, Taibai North Road, Beilin District, Xi’an, 710069 China; 2 School of Public Health, Tianjin Medical University, Qixiangtai Road, Heping District, Tianjin, 300070 China; 3 Nutritional Epidemiology, Department of Clinical Sciences Malmö, Lund University, Jan Waldenströms Gata 35, 21428 Malmö, Sweden

**Keywords:** Ultra-processed foods, Plasma protein, Cardiovascular disease

## Abstract

**Background:**

We aim to examine the association between ultra-processed foods (UPF) consumption and cardiovascular disease (CVD) risk and to identify plasma proteins associated with UPF.

**Methods:**

This prospective cohort study included 26,369 participants from the Swedish Malmö Diet and Cancer Study, established in 1991–1996. Dietary intake was assessed using a modified diet history method, and UPF consumption was estimated using the NOVA classification system. A total of 88 selected CVD-related proteins were measured among 4475 subjects. Incident CVD (coronary heart disease and ischemic stroke) was defined as a hospital admission or death through registers. Cox proportional hazards regression models were performed to analyze the associations of UPF intake with risks of CVD. Linear regression models were used to identify the plasma proteins associated with UPF intake.

**Results:**

During 24.6 years of median follow-up, 6236 participants developed CVD, of whom 3566 developed coronary heart disease and 3272 developed ischemic stroke. The adjusted hazard ratio (95% confidence interval) in the 4th versus 1st quartile of UPF was 1.18 (1.08, 1.29) for CVD, 1.20 (1.07, 1.35) for coronary heart disease, and 1.17 (1.03, 1.32) for ischemic stroke. Plasma proteins interleukin 18, tumor necrosis factor receptor 2, macrophage colony-stimulating factor 1, thrombomodulin, tumor necrosis factor receptor 1, hepatocyte growth factor, stem cell factor, resistin, C–C motif chemokine 3, and endothelial cell-specific molecule 1 were positively associated with UPF after correcting for multiple testing.

**Conclusions:**

Our study showed that high UPF intake increased the risk of CVD and was associated with several protein biomarkers. Future studies are warranted to validate these findings and assess the potential pathways between UPF intake and CVD.

**Supplementary Information:**

The online version contains supplementary material available at 10.1186/s12916-023-03111-2.

## Background

Cardiovascular disease (CVD), which includes coronary heart disease (CHD) and stroke, is the leading cause of death worldwide, including in Sweden [1]. Given the enormous burden that CVD causes on individuals, families, communities, systems of care, and society at large [2], the identification of modifiable risk factors for CVD enables public health measures to prevent or delay cases of CVD. According to previous studies, dietary intake is an important risk factor for CVD [3, 4].

Ultra-processed foods (UPF) are usually defined as ready-to-eat or heat formulations made by assembling food substances, mostly commodity ingredients, and “cosmetic” additives through a series of industrial processes [5]. They are often characterized by poor nutritional value and high energy density with low fiber and micronutrient content, as well as high amounts of sodium, saturated and trans fats, and simple sugars [5, 6]. In addition, UPF usually contains a great diversity of additives, many of which have shown adverse effects on the vascular system in animals and in vitro studies [7–9]. Nevertheless, few studies have been carried out to assess the relationship between UPF consumption and CVD risk [10–12]. Recently, a study reported that the share of dietary energy coming from UPF is 41% for men and 44% for women in Sweden, which is the highest proportion in Europe [13]. However, no previous study has examined the association of UPF with CVD risk in Sweden. Thus, we aim to investigate the association between UPF consumption and CVD risk in a large Sweden cohort.

Despite these data, the biological mechanisms underlying the complex associations between UPF intake and CVD risk are not yet fully understood. Plasma proteins are relevant targets for mechanistic study because they regulate biological processes and are affected by environmental exposures and serve many functions in behaviors and disease [14–16]. Therefore, understanding the influence of UPF consumption on plasma proteins may help evaluate mediators and pathways between UPF consumption and CVD, leading to the identification of novel biomarkers for risk prediction and targets for intervention. We investigated the relationship between CVD-related plasma proteins, UPF consumption, and incident CVD.

## Methods

### Population

The Malmö Diet and Cancer Study (MDCS) is a large prospective cohort study on associations between diet and health outcomes. From 1991 to 1996, all citizens aged 45–73 years at study entry were recruited in Malmö, a city in southern Sweden. Detailed descriptions of the cohort and representability have been published previously [17]. A sub-cohort, the Malmö Diet and Cancer-Cardiovascular cohort (MDC-CC), consisting of a random sample of 6103 participants, were re-recruited during 1991–1994 [18]. All participants provided written informed consent. The study conformed to the ethical guidelines of the 1975 Declaration of Helsinki and was approved by the Lund University Ethical Committee (LU51/90, LU 2009/633, LU 2011/537 and LU 2012/762).

Among the 30,447 participants in the MDCS, we excluded those with missing information on covariates (*n* = 2213) and with a history of CVD (*n* = 829). We also excluded participants with implausible total energy intake (men with < 800 kcal/day or > 4200 kcal/day or women with < 600 kcal/day or > 3500 kcal/day) (*n* = 1036), leaving 26,369 individuals. For plasma proteins, the analysis was restricted to the MDC-CC participants. The available sample was 4475 for identifying the proteins associated with UPF intake. A flowchart is presented in Additional file 1: Fig. S1.

### Dietary assessment and calculation of UPF intake

At baseline, dietary intake was collected using a modified diet history method consisting of (1) a 7-day food diary for registering cooked meals and cold beverages; (2) a 168-item food-frequency questionnaire (FFQ) for assessment of consumption frequencies and portion sizes of regularly eaten foods that were not covered by the 7-day food diary; and (3) a 45–60-min interview to ask for cooking methods and usual portion sizes for foods recorded in the food diary and to check for overlap between intakes reported by the food diary and the FFQ. Each food item and ingredient in the food diary was coded and summarized into the average amount of food consumed (g) per day. Information from the FFQ on portion sizes and frequencies was entered into the computer and converted into grams. All food information from the two sources was summarized into the average daily consumption of food groups (grams per day) for each individual. The food items were aggregated and categorized into 107 food groups. Energy and nutrient intakes were computed from the Malmö Diet and Cancer Food and Nutrient Database originated from PC KOST2-93, based on the Swedish National Food Agency. The validity of the diet history method has previously been examined with 18 days of weighted food records in a random sample of the Malmö population in 1984–1985. The validation study included 206 Malmö residents (101 men and 105 women) in the age range 50–69 years [19]. Energy-adjusted correlations (men/women) were for total fat 0.64/0.69, saturated fatty acids 0.56/0.68, bread 0.50/0.58, and cereals 0.74/0.73.

The food groups (except for alcohol beverages) were categorized into four NOVA food groups according to the extent and purpose of the industrial processing they undergo [5]: (1) unprocessed or minimally processed foods, e.g., fresh or dried fruits, fresh juice, vegetables, eggs, milk, and unprocessed meat; (2) processed culinary ingredients, e.g., butter and sugar; (3) processed foods, e.g., cheese, canned fish, high-fiber bread, and fried potato; (4) UPF, e.g., soft drinks, sweets/candies, chocolate, snacks, cookies, cakes, low-fiber bread, ice cream, sausage, and bacon. In this study, we focus on the fourth NOVA group. The example of foods in each category were listed in Additional file 1: Table S1.

### Plasma protein quantification

Blood samples were stored at − 80 °C after collection at baseline. Proteomic profiling was performed using the Olink platform (Olink Proteomics, Uppsala, Sweden) (https://www.olink.com/content/uploads/2015/12/0696-v1.3-Proseek-Multiplex-CVD-I-Validation-Data_final.pdf). A total of 92 selected CVD-related proteins were measured using the Olink proximity extension assay technology (CVD-I panel, Olink Proteomics, Uppsala, Sweden). Values were expressed as normalized protein expression values as arbitrary units on a log2 scale. For statistical analysis, we excluded four proteins that were available in less than 75% of the individuals in the present study sample (BetaNGF, ENRAGE, Interleukin-4, Brain natriuretic peptide 32). Hence, 88 proteins were available for the analysis.

### Outcome

All participants were followed up from baseline examination to the diagnosis of CVD, emigration from Sweden, death, or December 31, 2018, whichever came first. Total CVD were obtained through the Swedish Hospital Discharge Register [20], the Swedish Cause of Death Register, and the Stroke Register of Malmö [21]. Total CVD was defined as a hospital admission or death using International Classification of Diseases (ICD) codes, including coronary heart disease (CHD) (ICD-9 codes 410A-410X and ICD-10 code I21), death attributable to ischemic heart disease (ICD-9 codes: 410–414; ICD-10 codes: I20-I25), and ischemic stroke (ICD-9 code 434 and ICD-10 code I63).

### Assessment of covariates

Information on age and sex was collected by the Swedish personal identification number. Smoking habits (current, former, or never), educational level (the highest qualification attained), and other lifestyle factors were obtained using a structured questionnaire. The heredity score was constructed based on the participants’ self-reported family history of myocardial infarction and stroke (mother, father, or sibling with disease). Participants were categorized as 0: no heredity or no answer in questionnaire; 1–3: heredity from father, mother, and brother/sister, respectively, contributing with one “point” each. Leisure-time physical activity (LTPA) was assessed using questionnaire items adapted from the Minnesota Leisure Time Physical Activity Questionnaire. The total LTPA volume was expressed as the weekly metabolic equivalent hours (MET-hour/week). Alcohol consumption was divided into six categories. Zero consumers reported no consumption of alcohol in the 7-day food diary or during the previous year in the questionnaire. The other individuals were divided into sex-specific quintiles based on the reported alcohol intake from the 7-day food diary. Anthropometric measures were objectively assessed during physical examination by trained personnel. Body mass index (BMI) was calculated as kg/m^2^. Systolic and diastolic blood pressures were measured after 10 min of rest in the supine position. Hypertension (HBP) was defined as systolic/diastolic blood pressures ≥ 140/90 mmHg and/or current use of antihypertensive medications. The variable for “method” was created based on the interview time of 60 min before August 1994 and 45 min from 1 September 1994 [22]. A categorical variable (season) was created based on the season during which the data were collected. A diet quality index based on the Swedish dietary guidelines was calculated using the following factors: fiber (> 10 g/1000 kcal), fruit and vegetables (> 400 g/day), fish (> 300 g/week), added sugar (< 10% energy), and red meat (< 500 g/week). Each point was given for each favorable diet factor, with the total score ranging from 0 to 5 [23]. Energy misreporters were defined as participants who have a ratio of energy intake to basal metabolic rate outside the 95% confidence interval (CI) limits of the calculated physical activity level [22]. The “substantial change in dietary habits in the past” was derived from the questionnaire item ‘Have you substantially changed your eating habits because of illness or some other reasons?’ and dichotomized into one variable as non-changers and changers.

### Statistical analyses

Baseline characteristics are presented as the number (percentage) for categorical variables and the mean (standard deviation (SD)) for continuous variables. We used *t* tests or *χ*^2^ tests to examine participant characteristics according to participants’ sex-specific quartile of UPF consumption. The associations between baseline UPF consumption (as a continuous variable or quarters with sex-specific cut-offs) and CVD risk were explored using Cox proportional hazard models to calculate the hazard ratios (HRs) and 95% CIs. Covariates were adjusted for multivariable models. In model 1, we adjusted for age and sex; in model 2, we further included education, smoking status, alcohol consumption, LTPA, method, season, HBP, heredity score, total energy intake, and diet quality index; in model 3 (full model), we additionally adjusted for BMI. We also included restricted cubic spline term for UPF with five knots at 10th, 25th, 50th, 75th, and 90th centiles in model 3 to explore potential nonlinear relations of UPF consumption to CVD risk. The nonlinearity *P* value was estimated with a likelihood ratio test. The SAS macro named “%RCS_REG” was used to perform the restricted cubic spline analysis [24]. We constructed a directed acyclic graph (DAG) to justify the inclusion of the confounders using the program, DAGitty [25]. Additional file 1: Fig. S2 shows the DAG was derived from previous literature and expert knowledge.

The effect of substituting 1 standard deviation (equivalent to 211 g/day) UPF with an equivalent weight of unprocessed or minimally processed foods was estimated by including both terms as continuous variables in the same multiple regression model. The difference in the *β* coefficients for change in UPF and change in the unprocessed or minimally processed foods was used to estimate the HR; the corresponding variances and covariance were used to estimate 95% CI [26].

Linear regression models were used to identify the plasma proteins associated with UPF intake, adjusted for age and sex. We used the Bonferroni method to account for multiple testing. *P* < 0.05/88 for plasma proteins were deemed as statistically significant. In prospective analyses of associations of protein levels with risk of CVD, Cox proportional hazards models were used to estimate HRs of CVD per 1-SD higher protein markers adjusted for age and sex.

We performed stratified analyses to assess potential modification effects according to sex (female or male), age (≤ 60 or > 60 years), BMI (< 24.9 or ≥ 24.9 kg/m^2^), smoking status (never, former, and current), diet quality index (≤ 2 or > 2), LTPA (< 7.5, 7.5–15, 15–25, 25–50, and > 50 MET-hour/week), and HBP (yes or no). The interactions between these stratified covariates and UPF consumption were examined using the likelihood ratio test.

We conducted the following sensitivity analyses: (1) to consider the other NOVA groups, we examined the association between the percentage of UPF weight in the total food and the risk of CVD; (2) to consider total energy intake, we used energy-adjusted UPF (g/1000 kcal) as an alternate unit to compare results with UPF weight; (3) to reduce potential reverse causation, we excluded potential energy misreporters (i.e., non-adequate reporters of energy), those who indicated a substantial change in dietary habits in the past or events occurring during the first 2 years of follow-up to reduce potential reverse causation.

All analyses were performed using SAS software, version 9.4 (SAS Institute Inc., Cary, NC, USA). A two-sided *P* < 0.05 was set as the threshold for statistical significance. The reporting of this paper was based on the Strengthening the Reporting of Observational Studies in Epidemiology-Nutritional Epidemiology (STROBE-nut) guidelines, an Extension of the STROBE Statement [27].

## Results

During 551,124 person-years of follow-up (24.6 years of median follow-up), 6236 participants developed incident CVD. At baseline, the median UPF intake in the total population, in men, and in women was 348 g/day, 423 g/day, and 308 g/day, respectively. Baseline characteristics across quartiles of UPF consumption are given in Table 1. Higher quartiles of UPF consumption included people with the following characteristics: higher BMI, diastolic blood pressure, total energy intake, higher proportion of non-smokers, lower education level, lower alcohol intake, and lower diet quality index.

**Table 1:** Baseline characteristics of study population according to ultra-processed food consumption (*n* = 26,369)

Characteristics	All participants	Quartiles of sex-specific ultra-processed food consumption^a^			
		1st quartile (*n* = 6526)	2nd quartile (*n* = 6524)	3rd quartile (*n* = 6524)	4th quartile (*n* = 6526)
Age (years)	58 ± 7.62	58.2 ± 7.65	58.2 ± 7.67	58 ± 7.59	57.6 ± 7.57
Sex (men, %)	37.7	37.7	37.7	37.7	37.7
BMI (kg/m^2^)	25.7 ± 3.96	25.9 ± 3.92	25.5 ± 3.77	25.6 ± 3.92	25.8 ± 4.22
SBP (mmHg)	141.1 ± 20	141.3 ± 20.4	140.9 ± 19. 9	141 ± 19.9	141.1 ± 19.9
DBP (mmHg)	85.5 ± 9.95	85.7 ± 10.06	85.5 ± 9.81	85.4 ± 9.87	85.6 ± 10.0
Total energy intake (kcal/day)	2252.2 ± 604.1	1818.5 ± 449.1	2141 ± 455.6	2391.4 ± 519.9	2658.1 ± 628.8
LTPA (> 25 MET-hour/week, %)	52.6	52.0	52.7	53.0	52.6
University degree (%)	32.0	36.1	33.5	31.1	27.5
Alcohol consumption (%)					
Zero	6.20	6.13	5.05	6.08	7.55
Quintile 1	18.5	16.5	16.6	17.7	23.1
Quintile 2	18.7	16.7	18.3	19.5	20.4
Quintile 3	19.0	17.6	20.0	19.9	18.2
Quintile 4	18.8	20.1	20.3	19.1	15.9
Quintile 5	18.8	23.0	19.7	17.7	14.8
Smoking status (%)					
Smoker	28.1	28.7	27.7	27.3	28.7
Ex-smoker	33.2	35.7	33.6	32.2	31.5
Non-smoker	38.7	35.7	38.7	40.5	39.8
Hypertension (%)	60.8	61.1	60.2	61.4	60.3
Heredity score (> 0, %)					
Myocardial infarction	37.3	37.6	37.0	38.0	36.6
Stroke	26.9	26.4	27.3	26.9	26.9
Diet quality index	1.94 ± 1.29	2.46 ± 1.31	2.06 ± 1.25	1.82 ± 1.22	1.44 ± 1.16

*BMI* body mass index, *DBP* diastolic blood pressure, *LTPA* leisure-time physical activity, *MET* metabolic equivalent, *SBP* systolic blood pressure

^a^Continuous variables are expressed as means ± standard deviations and categorical variables are expressed as percentages

The associations between UPF consumption and risk of CVD were analyzed in three adjustment models (Table 2). After adjustment for multiple potential confounders, participants in the highest UPF consumption quartile had a greater risk of developing total CVD (HR: 1.18; 95% CI: 1.08–1.29), CHD (HR: 1.20; 95% CI: 1.07–1.35), and ischemic stroke (HR: 1.17; 95% CI: 1.03–1.32) compared to those in the lowest quartile. For each additional SD in UPF consumption (equivalent to 211 g/day), we observed increased risks of total CVD (HR = 1.07 (95% CI: 1.04–1.11); *P* < 0.001), CHD (HR = 1.07; 95% CI: 1.03–1.11; *P* < 0.001), and ischemic stroke (HR = 1.08; 95% CI:1.04–1.13); *P* < 0.001). The intake of UPF and risks of total CVD, CHD, and ischemic stroke were also presented by the restricted cubic spline (Additional file 1: Fig. S3).

**Table 2:** Associations between intake of ultra-processed food and cardiovascular disease (*n* = 26,369)^a^

	**Quartiles of sex-specific ultra-processed food consumption**^**b**^				***P* trend**	**Per SD increase**^**c**^	***P* value**
	**1st quartile**	**2nd quartile**	**3rd quartile**	**4th quartile**			
**Number**	6,593	6,592	6,591	6,593			
**Median ultra-processed food (g/day)**	199.7	293.8	397.9	620.5			
**CVD**							
Cases	1,546	1,553	1,512	1,625			
Person-years	136,479	138,352	139,752	136,539			
Mode1 1^d^	1 (reference)	0.98 (0.91, 1.05)	0.95 (0.89, 1.02)	1.09 (1.02, 1.17)	0.03	1.05 (1.02, 1.08)	0.0002
Mode1 2^e^	1 (reference)	1.03 (0.95, 1.11)	1.02 (0.94, 1.11)	1.20 (1.10, 1.32)	< 0.01	1.08 (1.05, 1.11)	< 0.001
Mode1 3^f^	1 (reference)	1.03 (0.95, 1.11)	1.02 (0.94, 1.10)	1.18 (1.08, 1.29)	< 0.01	1.07 (1.04, 1.11)	< 0.001
**CHD**							
Cases	898	884	859	925			
Person-years	141,192	142,945	144,431	141,664			
Mode1 1	1 (reference)	0.96 (0.87, 1.05)	0.93 (0.85, 1.02)	1.06 (0.97, 1.16)	0.32	1.04 (1.00, 1.07)	0.04
Mode1 2	1 (reference)	1.04 (0.94, 1.14)	1.04 (0.93, 1.16)	1.23 (1.09, 1.39)	< 0.01	1.08 (1.04, 1.12)	< 0.001
Mode1 3	1 (reference)	1.04 (0.94, 1.14)	1.03 (0.92, 1.15)	1.20 (1.07, 1.35)	< 0.01	1.07 (1.03, 1.11)	< 0.001
**Ischemic stroke**							
Cases	794	823	792	863			
Person-years	141,124	143,235	144,436	141,635			
Mode1 1	1 (reference)	1.00 (0.91, 1.11)	0.97 (0.88, 1.07)	1.12 (1.02, 1.24)	0.04	1.06 (1.03, 1.10)	< 0.001
Mode1 2	1 (reference)	1.03 (0.93, 1.14)	1.00 (0.89, 1.12)	1.18 (1.04, 1.33)	0.02	1.09 (1.04, 1.13)	< 0.001
Mode1 3	1 (reference)	1.03 (0.93, 1.14)	1.00 (0.89, 1.12)	1.17 (1.03, 1.32)	0.03	1.08 (1.04, 1.13)	< 0.001

^a^Obtained by using multivariable Cox regression model

^b^Hazard ratios (95% confidence interval) (all such values)

^c^1 standard deviation (SD) equal to 211 g/day of UPF intake

^d^Model 1 was adjusted for age and sex

^e^Model 2 was additionally adjusted for education, smoking status, alcohol consumption, physical activity, season, method, HBP, heredity score, total energy intake, and diet quality index

^f^Model 3 was adjusted for the same variables as in model 2 and further for BMI

In substitution analysis, replacing 1 SD UPF with an equivalent weight of unprocessed or minimally processed foods was associated with a 6% lower risk of CVD (HR 0.94; 95% CI 0.91–0.97; *P* < 0.001), a 7% lower risk of CHD (HR 0.94; 95% CI 0.91–0.98; *P* < 0.01), and a 6% lower risk of ischemic stroke (HR 0.93; 95% CI 0.89–0.97; *P* < 0.001) (Additional file 1: Table S2).

The results were largely consistent when analyses were stratified by sex (*P* interaction = 0.62), age (*P* interaction = 0.06), BMI (*P* interaction = 0.86), smoking status (*P* interaction = 0.53), diet quality index (*P* interaction = 0.24), LTPA (*P* interaction = 0.65), and HBP (*P* interaction = 0.27) (Additional file 1: Fig. S4).

In the sensitivity analyses using the percentage of UPF weight in the total food weight, the risk of CVD was similar to the main results (Additional file 1: Fig. S5). When we used energy-adjusted UPF (g/1000 kcal), a similar association was found (Additional file 1: Table S3). There was also no substantial change when we excluded participants who developed CVD (*n* = 297) during the first 2 years of follow-up (Additional file 1: Table S4), nor after excluding misreporters or those who indicated a substantial change in dietary habits in the past (Additional file 1: Table S4).

**Fig. 1:**
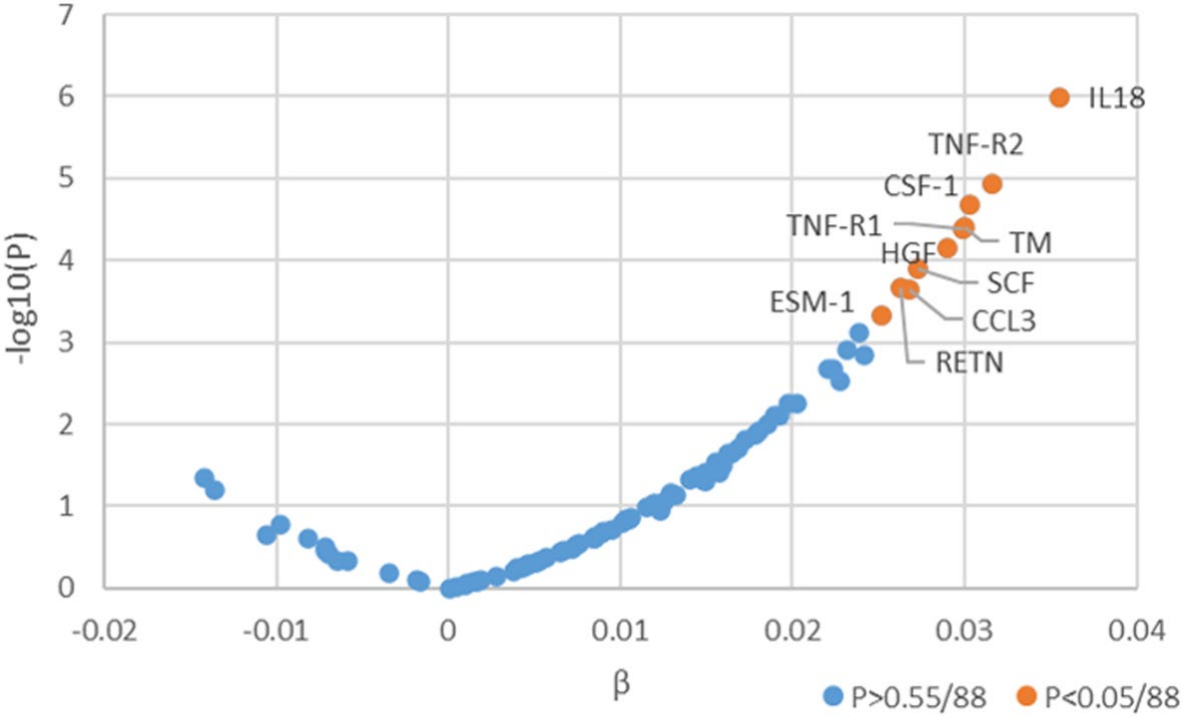
Volcano plot of association between UPF consumption and 88 plasma proteins in full sample analysis (*n* = 4475). Linear regressions were adjusted for age and sex. Orange, *P* < 0.05/88; Blue, *P* > 0.05/88

Figure 1 and Additional file 1: Table S5 show the plasma proteins associated with UPF intake. After correction for multiple testing and adjustment for age and sex, among the 88 plasma proteins, the concentration of ten proteins was found to be significantly higher among participants with higher consumption of UPF: interleukin 18 (IL18), tumor necrosis factor receptor 2 (TNF-R2), macrophage colony-stimulating factor 1 (CSF-1), thrombomodulin (TM), tumor necrosis factor receptor 1 (TNF-R1), hepatocyte growth factor (HGF), stem cell factor (SCF), resistin, C–C motif chemokine 3 (CCL3), and endothelial cell-specific molecule 1 (ESM-1).

## Discussion

In this population-based prospective cohort study, we found that UPF intake was positively associated with CVD risk. In addition, UPF intake was associated with a range of protein biomarkers, covering IL18, TNF-R2, CSF-1, TM, TNF-R1, HGF, SCF, resistin, CCL3 and ESM-1, which were also shown to be associated with risk of CVD.

Our findings on the relationship of UPF with CVD, CHD, and stroke are consistent with those from the Framingham Offspring [11], the French NutriNet-Santé [12], and the UK Biobank cohort studies [10]. Of note, we additionally included substitution analysis that explicitly compared UPF with unprocessed or minimally processed foods while accounting for total energy intake. Our substitution analysis showed that replacing UPF with unprocessed or minimally processed foods was associated with a lower risk of CVD. Similar to our results, a recent cohort study showed that substitution of 10% UPF weight in diet with an equivalent proportion of unprocessed or minimally processed foods was estimated to be associated with a 14% lower risk of mortality [28]. These findings collectively highlighted that consumption of unprocessed or minimally processed foods could be a healthy alternative when compared with food categorized as ultra-processed using the NOVA classification.

We identified ten proteins, IL18, TNF-R2, CSF-1, TM, TNF-R1, HGF, SCF, resistin, CCL3, and ESM-1, for which plasma levels were found to be significantly higher among participants with higher consumption of UPF. Some of these proteins have been found to be associated with compounds often found in foods categorized as ultra-processed. For example, a cross-sectional study has reported a positive association between sTNFR-2 concentration and trans-fat [29]. During the 1990s, before the reduction of trans fat in foods, trans fat could be found in for example biscuits and margarine. In addition, a cross-sectional study observed not only a positive association of resistin with saturated fat intake, but also an inverse association with Mediterranean diet [30]. Furthermore, experimental evidence from animal studies indicated that fructose and artificial sweeteners intake significantly increased plasma resistin [31, 32]. Artificial sweeteners and fructose, which is part of sucrose, are added to foods and beverages to make them sweet.

A higher intake of UPF might contribute to chronic inflammation by altering the production of beneficial bacterial metabolites such as short-chain fatty acids by the intestinal microbiota [33, 34]. It can also trigger oxidative stress and induce the transcription process of inflammatory genes, through the activation of the NF-κB and the innate immune system [35].

Plasma IL-18, TNFR2, and resistin play a regulatory role in inflammation [36–38], which is an underlying mechanism of CVD. Inflammation pathways may, therefore, represent a promising mechanism to explain the association between UPF consumption and CVD.

UPF are typically wrapped in plastic packages and may therefore contain endocrine-disrupting chemicals, such as bisphenol A, which have been associated with an increased risk of cardiometabolic outcomes [39]. An experiment study found that bisphenol A increased the production of inflammatory cytokines, including IL18 and tumor necrosis factor-α [40]. Moreover, chronic exposure to bisphenol A resulted in prominent inflammation and oxidative stress responses as evidenced by upregulation of IL-18 expression [41]. Our study showed that IL-18 was positively associated with UPF intake and associated with higher CVD risk.

Similar to our previous study, we found that participants with high levels of SCF have a lower risk of cardiovascular events [42]. SCF is involved in vasculogenesis and cardiac repair by stimulating the recruitment and activation of bone marrow-derived stem cells and tissue-resident progenitors [43]. An experiment study showed that bone marrow-derived stem cells activated through the SCF/c-kit pathway differentiate into vascular smooth muscle cells that contribute to vascular repair [44]. Although c-kit expressing smooth muscle cell could decrease the risk through protection of endothelial function and improved plaque stability, the associations between high levels of SCF and a lower risk of CVD events do not prove a causal relationship. Therefore, further study needs to investigate this association.

The major strengths of this study include the long duration of follow-up, large sample size, low loss to follow-up (< 1%), and the outcomes from high-quality national and local registers. Furthermore, this was the first proteomic analysis implicated in CVD of UPF consumption. The present study also has several potential limitations. First, dietary data were collected once at baseline, and follow-up period dietary changes were unaccounted for, which might have attenuated the power to detect an association. The bias of random measurement errors in estimating diet intake likely under- or overestimates the true association. However, a study showed the stability of dietary patterns over time [45]. Second, although the NOVA classification is the most widely used, misclassification bias cannot be ruled. For example, in the NOVA classification, bread can be classified as both processed food and UPF, depending on the packaging. We categorized whole grain breads as processed food and low-fiber breads as UPF. Third, the intake data has been collected including using a food diary which might be prone to underreporting. However, an extensive book of photographs (48 black and white photographs) was used to estimate the usual portion sizes of dishes and foods in the food diary. Besides, the combination of FFQ and food diary overcomes the shortcomings of two methods. Fourth, the ethnic homogeneity of the study may limit the generalizability of the findings to other nationalities. Fifth, the lack of an external validation cohort of the plasma proteins in relation to UPF intake is a potential concern. Sixth, the selected proteins associated with higher intake of UPF were only based on cross-sectional analyses; it is unlikely to infer causality. Finally, as in other observational studies, even though we have adjusted for a wide variety of covariates that relate to CVD, residual confounding by unidentified confounders is still possible.

## Conclusion

Our study showed higher UPF intake was associated with a higher risk of CVD, while substituting unprocessed or minimally processed foods for UPF was associated with lower risk of CVD. The plasma proteomic analysis of UPF intake might provide clues to underlying biological mechanisms. Future studies are warranted to validate these findings and assess the potential pathways between UPF intake and CVD.

### Supplementary Information

**Additional file 1:** **Table S1.** Examples of food products considered in each food category according to the NOVA classification. **Table S2.** Associations of substituting ultra-processed foods (g/day) with unprocessed or minimally processed foods in relation to incident cardiovascular disease. **Table S3.** Associations between intake of energy adjusted ultra-processed food ((g/1000 kcal) and cardiovascular disease. **Table S4.** Sensitivity analysis for associations of ultra-processed food consumption with cardiovascular disease. **Table S5. **Plasma proteins associated with UPF intake. **Table S6. **Association between plasma protein and CVD. **Fig. S1**. Flowchart of participant selection from the Malmö Diet and Cancer Study. **Fig. S2. **Directed acyclic graph (DAG) derived from previous literature and expert knowledge. **Fig. S3**. Restricted cubic spline plots to assess association between UPF consumption and CVD. **Fig. S4**. Association between UPF intake and incident CVD among different subgroups. **Table S1.** Examples of food products considered in each food category according to the NOVA classification. **Table S2.** Associations of substituting ultra-processed foods (g/day) with unprocessed or minimally processed foods in relation to incident cardiovascular disease. **Table S3.** Associations between intake of energy adjusted ultra-processed food ((g/ 1000 kcal) and cardiovascular disease. **Table S4.** Sensitivity analysis for associations of ultra-processed food consumption with cardiovascular disease. **Table S5. **Plasma proteins associated with UPF intake. **Table S6. **Association between plasma protein and CVD. **Fig. S1**. Flowchart of participant selection from the Malmö Diet and Cancer Study. **Fig. S2. **Directed acyclic graph (DAG) derived from previous literature and expert knowledge. **Fig. S3**. Restricted cubic spline plots to assess association between UPF consumption and CVD. **Fig. S4**. Association between UPF intake and incident CVD among different subgroups. **Figure S5**. Associations between intake of ultra-processed food proportion and cardiovascular disease.

## Data Availability

The data that support the findings of this study are available from the Malmö Population-Based Cohorts Joint Database but restrictions apply to the availability of these data, which were used under license for the current study, and so are not publicly available. Data are however available from the authors upon reasonable request and with permission of the Malmö Population-Based Cohorts Joint Database.

## Abbreviations

BMI: Body mass index
CCL3: C-C motif chemokine 3
CI: Confidence interval
CHD: Coronary heart disease
CSF-1: Colony stimulating factor 1
CVD: Cardiovascular disease
DAG: Directed acyclic graph
ESM-1: Endothelial cell-specific molecule 1
FFQ: Food frequency questionnaire
HBP: Hypertension
HR: Hazard ratio
HGF: Hepatocyte growth factor
ICD: International Classification of Diseases
IL18: Interleukin-18
LTPA: Leisure-time physical activity
MDCS: The Malmö Diet and Cancer Study
MDC-CC: The Malmö Diet and Cancer-Cardiovascular Cohort
MET: Metabolic equivalent task
SD: Standard deviation
SCF: Stem cell factor
TNF-R1: Tumor necrosis factor receptor 1
TNF-R2: Tumor necrosis factor receptor 2
TM: Thrombomodulin
UPF: Ultra-processed food
