# Supplementary material for: Associations of ultra-processed food consumption, circulating protein biomarkers, and risk of cardiovascular disease
Source: BMC Med. 2023 Nov 3;21:415. doi: 10.1186/s12916-023-03111-2 (PMC10623817; doi:10.1186/s12916-023-03111-2)
Supplement: Supplementary file 1 — Additional file 1: Table S1. Examples of food products considered in each food category according to the NOVA classification. Table S2. Associations of substituting ultra-processed foods (g/day) with unprocessed or minimally processed foods in relation to incident cardiovascular disease. Table S3. Associations between intake of energy adjusted ultra-processed food ((g/1000 kcal) and cardiovascular disease. Table S4. Sensitivity analysis for associations of ultra-processed food consumption with cardiovascular disease. Table S5. Plasma proteins associated with UPF intake. Table S6. Association between plasma protein and CVD. Fig. S1. Flowchart of participant selection from the Malmö Diet and Cancer Study. Fig. S2. Directed acyclic graph (DAG) derived from previous literature and expert knowledge. Fig. S3. Restricted cubic spline plots to assess association between UPF consumption and CVD. Fig. S4. Association between UPF intake and incident CVD among different subgroups. Table S1. Examples of food products considered in each food category according to the NOVA classification. Table S2. Associations of substituting ultra-processed foods (g/day) with unprocessed or minimally processed foods in relation to incident cardiovascular disease. Table S3. Associations between intake of energy adjusted ultra-processed food ((g/ 1000 kcal) and cardiovascular disease. Table S4. Sensitivity analysis for associations of ultra-processed food consumption with cardiovascular disease. Table S5. Plasma proteins associated with UPF intake. Table S6. Association between plasma protein and CVD. Fig. S1. Flowchart of participant selection from the Malmö Diet and Cancer Study. Fig. S2. Directed acyclic graph (DAG) derived from previous literature and expert knowledge. Fig. S3. Restricted cubic spline plots to assess association between UPF consumption and CVD. Fig. S4. Association between UPF intake and incident CVD among different subgroups. Figure S5. Associations between intake of ultra-pr [file 12916_2023_3111_MOESM1_ESM.docx]

| **Table S1.** Examples of food products considered in each food category according to the NOVA classification | |
| --- | --- |
| **Food category** | **Food items** |
| Unprocessed or minimally processed foods | Vegetables, vegetable juice, fruits, fruit juice, potato, cereals/flakes without sugar, flour, rice, egg, unprocessed meat (pork, lamb, poultry, game meat, fish, shellfish), milk, fermented milk, cream, coffee, tea, water, nuts, soy |
| Processed culinary ingredients | Oil/lard, butter, sugar |
| Processed foods | Cereals/flakes>15% fiber with added sugar, canned fish and herring, cheese, vinegar, spices, broth, fried potato, high-fiber bread |
| Ultra-processed foods | Soft drinks, sweets/candies, chocolate, snacks, cookies, cakes, ice cream, margarine, mayonnaise, wafers, wheat crusts, low-fiber bread, marmalade, jam and honey, gruel, breakfast cereals, tomato ketchup, nutritional powder, industrial soups, fish sticks, sausage, bacon and ham , blood pudding |

| **Table S2.** Associations of substituting ultra-processed foods (g/day) with unprocessed or minimally processed foods in relation to incident cardiovascular disease. | | |
| --- | --- | --- |
|  | **Substituting 1 SD ultra-processed foods with unprocessed or minimally processed foods** | |
|  | **HR (95% CI) ^a^** | ***P* value** |
| **CVD** | 0.94 (0.91, 0.97) | <0.001 |
| **CHD** | 0.94 (0.91, 0.98) | <0.01 |
| **Ischemic stroke** | 0.93 (0.89, 0.97) | <0.001 |
| ^a^ Adjusted for age, sex, education, smoking status, alcohol consumption, physical activity, season, method, HBP, heredity score, total energy intake, diet quality index, and BMI. | | |

| **Table S3.** Associations between intake of energy adjusted (g/ 1000 kcal) ultra-processed food and cardiovascular disease ^a^. | | | | | |
| --- | --- | --- | --- | --- | --- |
|  | **Quartiles of ultra-processed food consumption ^b^** | | | | ***P* trend** |
|  | **1st quartile** | **2nd quartile** | **3rd quartile** | **4th quartile** |  |
| **Number** | 6,593 | 6,592 | 6,591 | 6,593 |  |
| **CVD** |  |  |  |  |  |
| Cases | 1,465 | 1,525 | 1,570 | 1,676 |  |
| Person-years | 139,334 | 139,575 | 137,773 | 134,442 |  |
| Model 1 ^c^ | 1 (reference) | 0.98 (0.91, 1.06) | 1.02 (0.95, 1.10) | 1.14 (1.06, 1.22) | <0.001 |
| Model 2 ^d^ | 1 (reference) | 0.98 (0.91, 1.06) | 1.03 (0.95, 1.11) | 1.14 (1.05, 1.23) | <0.001 |
| Model 3 ^e^ | 1 (reference) | 0.98 (0.91, 1.06) | 1.03 (0.95, 1.10) | 1.12 (1.04, 1.21) | <0.01 |
| **CHD** |  |  |  |  |  |
| Cases | 831 | 879 | 880 | 976 |  |
| Person-years | 139,334 | 139,575 | 137,773 | 134,442 |  |
| Model 1 | 1 (reference) | 1.01 (0.91, 1.11) | 1.01 (0.92, 1.11) | 1.16 (1.06, 1.28) | <0.01 |
| Model 2 | 1 (reference) | 1.01 (0.92, 1.12) | 1.02 (0.92, 1.13) | 1.17 (1.06, 1.30) | <0.01 |
| Model 3 | 1 (reference) | 1.01 (0.92, 1.12) | 1.02 (0.92, 1.12) | 1.15 (1.04, 1.27) | <0.01 |
| **Ischemic stroke** |  |  |  |  |  |
| Cases | 770 | 798 | 841 | 863 |  |
| Person-years | 139,334 | 139,575 | 137,773 | 134,442 |  |
| Model 1 | 1 (reference) | 0.97 (0.88, 1.07) | 1.03 (0.94, 1.14) | 1.10 (1.00, 1.22) | 0.02 |
| Model 2 | 1 (reference) | 0.97 (0.88, 1.07) | 1.04 (0.94, 1.15) | 1.10 (0.99, 1.22) | 0.04 |
| Model 3 | 1 (reference) | 0.97 (0.88, 1.07) | 1.04 (0.93, 1.15) | 1.09 (0.98, 1.21) | 0.06 |
| ^a^ Obtained by using multivariable Cox regression model. | | | | | |
| ^b^ Hazard ratios (95% confidence interval) (all such values). | | | | | |
| ^c^ Model 1 was adjusted for age and sex. | | | | | |
| ^d^ Model 2 was additionally adjusted for education, smoking status, alcohol consumption, physical activity, season, method, HBP, heredity score, total energy intake and the diet quality index. | | | | | |
| ^e^ Model 3 was adjusted for the same variables as in model 2 and further for BMI. | | | | | |

| **Table S4.** Sensitivity analysis for associations of ultra-processed food consumption with cardiovascular disease ^a^. | | | | | | | | | | | | | | |
| --- | --- | --- | --- | --- | --- | --- | --- | --- | --- | --- | --- | --- | --- | --- |
|  | Excluding of the first 2 years of follow-up | | | Excluding of misreporters | | | Excluding of a substantial change in dietary habits | | | | | | | |
|  | Cases/non-cases | HR (95% CI) ^b^ | *P* value | Cases/non-cases | HR (95% CI) | *P* value | | | Cases/non-cases | | | HR (95% CI) | *P* value | |
| **CVD** | 5938/20134 | 1.07 (1.04, 1.11) | <0.001 | 5186/16394 | 1.08 (1.04, 1.11) | | | <0.001 | | 4590/15585 | 1.06 (1.02, 1.09) | | | <0.01 |
| **CHD** | 3368/22704 | 1.07 (1.03, 1.11) | <0.01 | 2970/18610 | 1.08 (1.03, 1.12) | | | <0.01 | | 2574/17601 | 1.05 (1.00, 1.10) | | | 0.06 |
| **Ischemic stroke** | 3119/22953 | 1.08 (1.04, 1.13) | <0.01 | 2711/18869 | 1.09 (1.04, 1.14) | | | <0.01 | | 2443/17732 | 1.07 (1.02, 1.13) | | | <0.01 |
| ^a^ Adjusted for age, sex, education, smoking status, alcohol consumption, physical activity, season, method, HBP, heredity score, total energy intake, diet quality index and BMI. | | | | | | | | | | | | | | |

^b^ Values are hazard ratios (95% confidence interval) for per one standard deviation increase unless otherwise indicated.

| **Table S5.** Plasma proteins associated with UPF intake (n=4,475) ^a, b^. | | | | |
| --- | --- | --- | --- | --- |
| **Proteins** | **β** | ***P* value ^c^** | **95% confidence interval** | |
| IL-18 | 0.035565911 | 1.02196E-06 | 0.02131901 | 0.049812812 |
| TNF-R2 | 0.031640289 | 1.18326E-05 | 0.01749613 | 0.045784448 |
| CSF-1 | 0.030345924 | 2.09861E-05 | 0.016376097 | 0.04431575 |
| TM | 0.03000327 | 3.8361E-05 | 0.015730673 | 0.044275867 |
| TNF-R1 | 0.029903842 | 4.20784E-05 | 0.015604163 | 0.044203521 |
| HGF | 0.029029921 | 6.95975E-05 | 0.014735206 | 0.043324637 |
| SCF | 0.027336093 | 0.000124128 | 0.013384148 | 0.041288037 |
| RETN | 0.026319891 | 0.000217715 | 0.01237604 | 0.040263743 |
| CCL3 | 0.026840752 | 0.000229703 | 0.012568386 | 0.041113118 |
| ESM-1 | 0.025181815 | 0.000461778 | 0.011094954 | 0.039268677 |
| CCL20 | 0.023909429 | 0.000779486 | 0.009968238 | 0.037850621 |
| CCL4 | 0.023165421 | 0.001251537 | 0.009100166 | 0.037230677 |
| MB | 0.024174589 | 0.00145004 | 0.009301102 | 0.039048077 |
| U-PAR | 0.022038871 | 0.002139753 | 0.007973638 | 0.036104104 |
| CTSL1 | 0.022405379 | 0.002143497 | 0.008103805 | 0.036706952 |
| IL-1ra | 0.022805005 | 0.003002888 | 0.007747391 | 0.03786262 |
| FAS | 0.02022405 | 0.005487031 | 0.005953098 | 0.034495002 |
| IL27-A | 0.019792498 | 0.005618208 | 0.005787262 | 0.033797734 |
| MPO | 0.018950903 | 0.007747625 | 0.005004947 | 0.032896859 |
| TRAIL-R2 | 0.019253398 | 0.008004424 | 0.005026062 | 0.033480734 |
| IL-16 | 0.018602439 | 0.009766079 | 0.004494746 | 0.032710132 |
| IL-8 | 0.018075738 | 0.012024069 | 0.003971102 | 0.032180374 |
| SPON1 | 0.017868896 | 0.013559718 | 0.003684227 | 0.032053565 |
| RAGE | 0.017285687 | 0.015628926 | 0.003272871 | 0.031298503 |
| SELE | 0.016873113 | 0.019516719 | 0.002714454 | 0.031031772 |
| hK11 | 0.01628575 | 0.022974873 | 0.002249169 | 0.030322331 |
| CSTB | 0.016480999 | 0.02317625 | 0.002255255 | 0.030706744 |
| CX3CL1 | 0.015578429 | 0.02907865 | 0.001588635 | 0.029568222 |
| MMP-12 | 0.015899327 | 0.032095746 | 0.001360057 | 0.030438596 |
| MMP-7 | 0.014976199 | 0.038162672 | 0.000817685 | 0.029134713 |
| PAPPA | 0.015781746 | 0.03883441 | 0.000809907 | 0.030753586 |
| TRAIL | 0.014412252 | 0.043107252 | 0.000446993 | 0.028377511 |
| CXCL1 | -0.014216893 | 0.045195346 | -0.028129361 | -0.000304425 |
| PlGF | 0.014703568 | 0.047488045 | 0.000162969 | 0.029244167 |
| MMP-10 | 0.014056697 | 0.047644707 | 0.000145985 | 0.027967408 |
| GDF-15 | 0.014991478 | 0.049678213 | 2.1093E-05 | 0.029961863 |
| CHI3L1 | -0.013619 | 0.064268523 | -0.028045415 | 0.000807414 |
| TRANCE | 0.012969024 | 0.06932762 | -0.001026404 | 0.026964453 |
| VEGF-A | 0.012923222 | 0.071252291 | -0.001118774 | 0.026965218 |
| CA-125 | 0.013249086 | 0.074245008 | -0.001298425 | 0.027796596 |
| CTSD | 0.012573573 | 0.089128661 | -0.001923419 | 0.027070565 |
| LOX-1 | 0.011975337 | 0.092478229 | -0.001975932 | 0.025926605 |
| KLK6 | 0.011982952 | 0.094142411 | -0.002048328 | 0.026014231 |
| FGF-23 | 0.011557835 | 0.103800072 | -0.002368768 | 0.025484438 |
| FABP4 | 0.012306372 | 0.111947145 | -0.002869376 | 0.02748212 |
| AGRP | 0.010581715 | 0.139526032 | -0.003456109 | 0.024619538 |
| Dkk-1 | 0.010362549 | 0.144886392 | -0.003570572 | 0.02429567 |
| IL-6 | 0.010491694 | 0.145473016 | -0.003635662 | 0.02461905 |
| TIE2 | 0.010121614 | 0.156595554 | -0.003883628 | 0.024126856 |
| SRC | -0.009761883 | 0.169277112 | -0.023683024 | 0.004159257 |
| GAL | 0.009515099 | 0.194717575 | -0.004868181 | 0.023898379 |
| CD40 | 0.008971417 | 0.206886485 | -0.004961588 | 0.022904422 |
| ECP | 0.008892693 | 0.211389042 | -0.005055305 | 0.022840691 |
| MMP-3 | -0.010611344 | 0.225700495 | -0.027780569 | 0.00655788 |
| TNFSF14 | 0.008547516 | 0.22964619 | -0.005400274 | 0.022495305 |
| PRL | 0.008461999 | 0.242172347 | -0.00572052 | 0.022644519 |
| OPG | 0.008565641 | 0.244541315 | -0.005862932 | 0.022994213 |
| CD40-L | -0.008194402 | 0.248534882 | -0.022114823 | 0.005726019 |
| TF | 0.007652646 | 0.290711233 | -0.006545467 | 0.021850759 |
| MCP-1 | 0.007380863 | 0.303676073 | -0.006685567 | 0.021447294 |
| MMP-1 | -0.007180479 | 0.31288716 | -0.021128112 | 0.006767153 |
| PTX3 | 0.007184498 | 0.325341239 | -0.007134937 | 0.021503933 |
| ST2 | -0.007128038 | 0.35077897 | -0.022103207 | 0.007847131 |
| Gal-3 | 0.006736825 | 0.353940935 | -0.007509703 | 0.020983352 |
| FS | 0.006482872 | 0.368875455 | -0.007659805 | 0.02062555 |
| mAmP | -0.006976927 | 0.38804663 | -0.022822786 | 0.008868932 |
| HB-EGF | 0.005752376 | 0.418531996 | -0.008186892 | 0.019691645 |
| CXCL6 | 0.005322964 | 0.454156989 | -0.008617844 | 0.019263773 |
| LEP | -0.006481124 | 0.458742653 | -0.023628962 | 0.010666714 |
| PSGL-1 | -0.00585118 | 0.471449983 | -0.02178027 | 0.010077911 |
| SIRT2 | 0.005063868 | 0.475643969 | -0.008852416 | 0.018980151 |
| CASP-8 | 0.004713988 | 0.517294362 | -0.009557409 | 0.018985385 |
| ITGB1BP2 | 0.004646349 | 0.521606662 | -0.009566329 | 0.018859027 |
| CXCL16 | 0.004495711 | 0.529608877 | -0.009524482 | 0.018515904 |
| PECAM-1 | 0.004264671 | 0.550539275 | -0.009740186 | 0.018269527 |
| NEMO | 0.004013829 | 0.57227531 | -0.009920046 | 0.017947703 |
| AM | 0.003770081 | 0.606075403 | -0.010561769 | 0.018101931 |
| REN | -0.003449501 | 0.640782405 | -0.017941985 | 0.011042983 |
| t-PA | 0.002767028 | 0.704942809 | -0.01155834 | 0.017092395 |
| PDGF subunit B | 0.001870327 | 0.792577744 | -0.012072783 | 0.015813437 |
| EGF | -0.001837812 | 0.796033181 | -0.015776011 | 0.012100387 |
| HSP 27 | 0.001640805 | 0.817850537 | -0.012325559 | 0.015607168 |
| VEGF-D | 0.001456895 | 0.839081147 | -0.012607634 | 0.015521424 |
| NT-pro-BNP | -0.001589077 | 0.843831835 | -0.017403389 | 0.014225235 |
| IL-6RA | 0.001095785 | 0.877758613 | -0.012870411 | 0.01506198 |
| GH | 0.000954581 | 0.911619402 | -0.015904969 | 0.017814131 |
| PAR-1 | 0.000496461 | 0.944360498 | -0.013448945 | 0.014441868 |
| TIM | 9.72201E-05 | 0.989585589 | -0.014504042 | 0.014698482 |
| ^a^ Using multiple linear regression analysis with adjustment for age and sex. | | | | |
| ^b^ Proteins were standardized as z-scores.  ^c^ *P*<0.05/88 for plasma proteins were deemed as statistically significant. | | | | |

| **Table S6.** Association between plasma protein and CVD (n=4,475) ^a^. | | | | | | |
| --- | --- | --- | --- | --- | --- | --- |
| **Proteins** | **CVD** | | **CHD** | | **Ischemic stroke** | |
|  | HR (95% CI) ^b, c^ | *P* value | HR (95% CI) | *P* value | HR (95% CI) | *P* value |
| **IL18** | 1.13 (1.07, 1.20) | <0.001 | 1.12 (1.04, 1.21) | <0.01 | 1.15 (1.07, 1.25) | <0.001 |
| **TNF-R2** | 1.11 (1.05, 1.19) | <0.001 | 1.14 (1.05, 1.24) | <0.01 | 1.10 (1.01, 1.20) | 0.03 |
| **CSF-1** | 1.10 (1.03, 1.18) | <0.001 | 1.11 (1.02, 1.21) | 0.02 | 1.10 (1.01, 1.20) | 0.03 |
| **TM** | 1.05 (0.98, 1.12) | 0.14 | 1.04 (0.95, 1.13) | 0.41 | 1.06 (0.97, 1.16) | 0.22 |
| **TNF-R1** | 1.12 (1.05, 1.20) | <0.001 | 1.16 (1.06, 1.27) | <0.01 | 1.10 (1.00, 1.20) | 0.04 |
| **SCF** | 0.85 (0.80, 0.91) | <0.001 | 0.85 (0.78, 0.93) | <0.01 | 0.86 (0.79, 0.93) | <0.001 |
| **HGF** | 1.23 (1.15, 1.31) | <0.001 | 1.23 (1.13, 1.34) | <0.001 | 1.21 (1.11, 1.32) | <0.001 |
| **resistin** | 1.10 (1.03, 1.17) | <0.01 | 1.14 (1.05, 1.24) | <0.01 | 1.08 (0.99, 1.17) | 0.09 |
| **CCL3** | 1.12 (1.06, 1.19) | <0.001 | 1.10 (1.02, 1.19) | 0.02 | 1.14 (1.06, 1.23) | <0.001 |
| **ESM-1** | 0.94 (0.88, 1.00) | 0.04 | 0.90 (0.83, 0.98) | 0.02 | 0.95 (0.88, 1.04) | 0.28 |
| ^a^ CI, confidence interval; IL18, Interleukin 18; TNF-R2, tumor necrosis factor receptor 2; CSF-1, macrophage colonystimulating factor 1; TM, thrombomodulin; TNF-R1, tumor necrosis factor receptor 1; HGF, hepatocyte growth facto; SCF, stem cell factor; CCL3，C-C motif chemokine 3. | | | | | | |
| ^b^ Values are hazard ratios (95% confidence interval) for per one standard deviation increase unless otherwise indicated. | | | | | | |
| ^c^ Adjusted for age and sex. | | | | | | |

**Fig. S1**. Flowchart of participant selection from the Malmö Diet and Cancer Study.


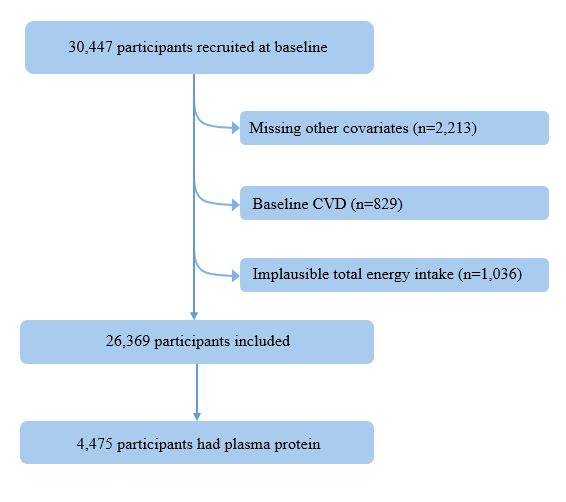


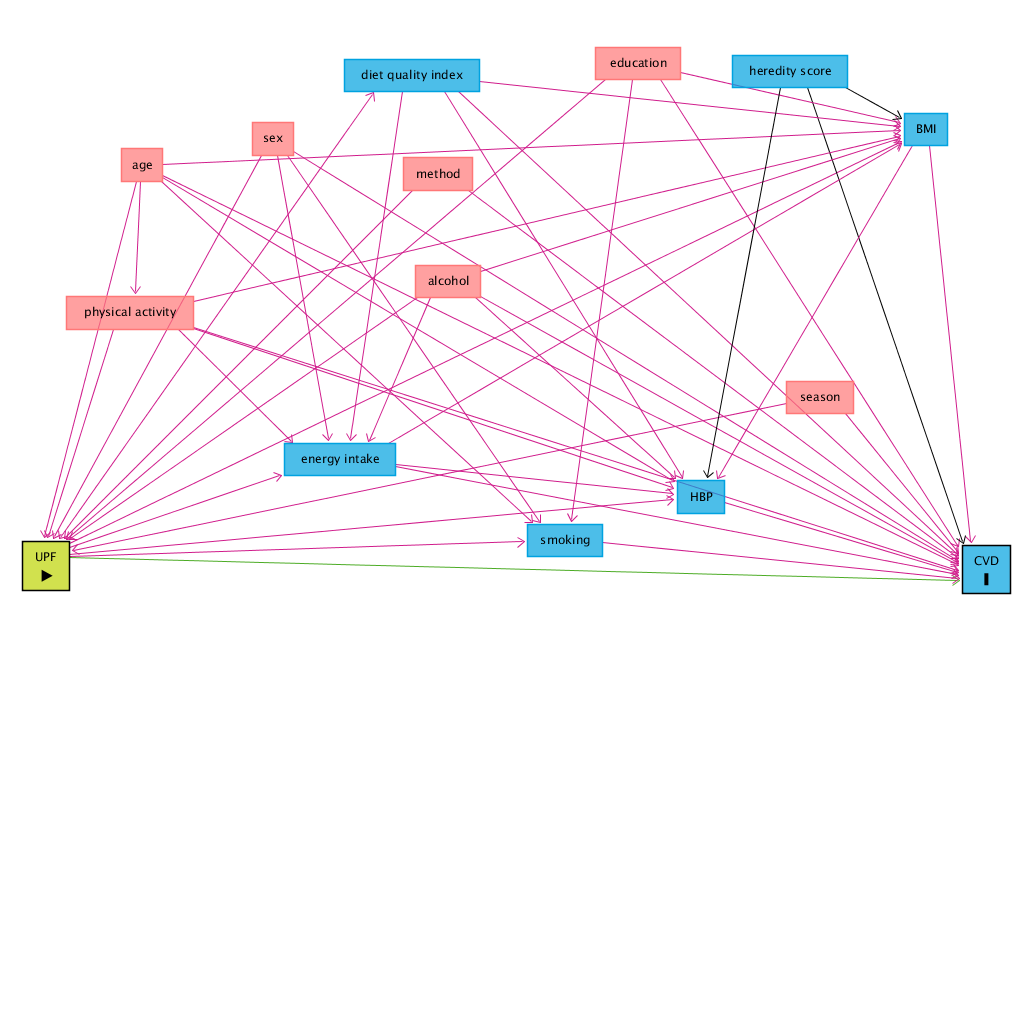
**Fig. S2.** Directed acyclic graph (DAG) derived from previous literature and expert knowledge.

Arrows represent causal associations.

UPF intake is exposure, and CVD is the outcome. BMI, body mass index; HBP, hypertension.

**Fig. S3**. Restricted cubic spline plots to assess association between UPF consumption and CVD ^a^.


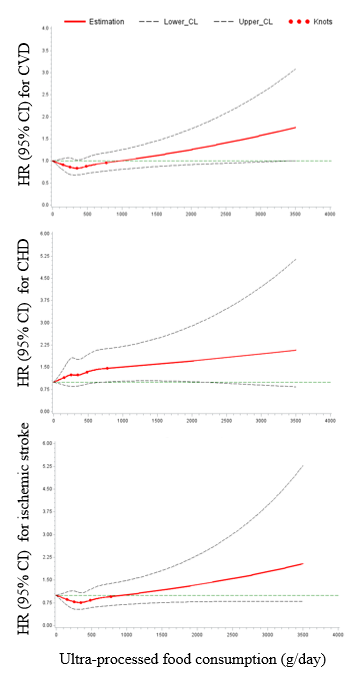


^a^ The HRs and 95% CIs above were adjusted for age, sex, education, smoking status, alcohol consumption, physical activity, season, method, HBP, heredity score, total energy intake, diet quality index and BMI. CVD, cardiovascular disease; CHD, coronary heart disease.

**Fig. S4**. Association between UPF intake and incident CVD among different subgroups ^a^.

^a^ Adjusted for age, sex, education, smoking status, alcohol consumption, physical activity, season, method, HBP, heredity score, total energy intake, diet quality index, and BMI.

^b^ Hazard ratio for per increase of 1 SD UPF consumption.

CVD, cardiovascular disease; CI, confidence interval; HR, hazards ratio; HBP, hypertension; UPF, ultra-processed food; LTPA, leisure-time physical activity; MET, metabolic equivalent

**Fig. S5**. Associations between intake of ultra-processed food proportion and cardiovascular disease.

^a^ Obtained by using multivariable Cox regression model.

^b^ Hazard ratio for an absolute increment of 10 in percentage of UPF in diet.

^c^ Model 1 was adjusted for age and sex.

^d^ Model 2 was additionally adjusted for education, smoking status, alcohol consumption, physical activity, season, method, HBP, heredity score, total energy intake, and diet quality index.

^e^ Model 3 was adjusted for the same variables as in model 2 and further for BMI.
